# Canadian 24-hour movement guidelines for the early years (0–4 years): exploring the perceptions of stakeholders and end users regarding their acceptability, barriers to uptake, and dissemination

**DOI:** 10.1186/s12889-017-4853-z

**Published:** 2017-11-20

**Authors:** Negin Riazi, Subha Ramanathan, Meghan O’Neill, Mark S. Tremblay, Guy Faulkner

**Affiliations:** 1 School of Kinesiology, University of British Columbia, 2146 Health Sciences Mall, Vancouver, BC V6T 1Z3 Canada; 2 Healthy Active Living and Obesity Research Group, CHEO Research Institute, 401 Smyth Road, Ottawa, ON K1H 8L1 Canada

**Keywords:** Guidelines, Early years, Infants, Toddlers, Preschoolers, Physical activity, Sedentary behaviour, Screen time, Sleep, Public health

## Abstract

**Background:**

It is important to engage stakeholders and end users in the development of guidelines for knowledge translation purposes. The aim of this study was to examine stakeholders’ (experts in pediatric and family medicine, physical activity knowledge translation, and research) and end users’ (parents and early childhood educators) perceptions of the Canadian 24-Hour Movement Guidelines for the Early Years (0–4 years).

**Methods:**

Stakeholders (*n* = 10) engaged in telephone interviews and end users (*n* = 92) participated in focus groups (*n* = 14) to discuss perceived clarity and need for the guidelines, potential barriers to implementation, identification of credible messengers, and methods for dissemination of the guidelines. A thematic analysis was conducted.

**Results:**

The proposed guidelines were very well received by both stakeholders and end users. A clear need for such guidelines was identified, and most believed the guidelines were achievable. Stakeholders and end users identified several potential barriers to uptake, including low awareness of current guidelines; ‘daily challenges’ such as allure of screen time, lack of time, and competing priorities; and challenges in the context of shifting social norms. A range of methods and messengers of dissemination were identified. Medical and child care settings were the most frequently cited places for dissemination, and physicians and early childhood educators were the most common suggestions for messengers.

**Conclusions:**

There was consistent support for the Canadian 24-Hour Movement Guidelines for the Early Years (0–4 years) from both stakeholders and end users. Moving forward, it is important to dedicate appropriate support and funding toward dissemination efforts in order to reach end users, particularly parents and early childhood educators.

## Background

According to the World Health Organization, physical inactivity in children has become a global epidemiological concern [1]. In Canada, data collected using accelerometers in the Canadian Health Measures Survey show that only 8% of 5- to 17-year-olds accumulate at least 60 min of moderate to vigorous physical activity [2]. The same World Health Organization report estimated that in 2016, 41 million children under the age of 5 years were considered overweight [1]. Physical inactivity combined with tripling obesity rates over the past three decades (from 5% to 15%) [3] and shifting social norms toward more sedentary activities raise concern for children’s health and well-being. Even society’s youngest generation (0–4 years) faces challenges with increasing rates of obesity and engagement in sedentary activities [4].

The early years (0–4 years) are a critical period for growth and development for infants, toddlers, and preschoolers. Although the benefits of physical activity for school-aged children have been well established [5], there has been less focus on the 0–4 age range. As described in this special issue, accumulating research demonstrates a positive relationship between higher levels of physical activity and positive health outcomes [6]. There is also increasing evidence to show the importance of minimizing screen time in the early years [7], getting good-quality sleep in these years [8], and showing how physical activity, sedentary behaviour, and sleep interact to produce optimal health benefits [9].

As toddlers and preschoolers transition into childhood and adolescence, physical activity tends to decrease [10] while sedentary behaviours, such as watching television, are introduced into the daily schedule [11]. It is therefore important to help children develop healthy lifestyle habits early on, and promoting a physically active lifestyle at an early age may help children carry these healthy habits into adulthood [12]. A preliminary step to combatting the complex issues of physical inactivity and increasing sedentary behaviours is the development of guidelines. These provide evidence-based recommendations and standards that have the potential to contribute significantly to overall health, and can also be used for surveillance purposes [13]. Engaging stakeholders and end users is a crucial step, as this can help to ensure effective knowledge translation [14], determine if guidelines are relevant to the stakeholders’ and end users’ needs [15], and identify strategies for effectively communicating the guidelines [16].

Limited research has examined perceptions of physical activity and sedentary behaviour guidelines for children’s early years. In the United Kingdom, in-depth interviews were conducted with mothers of preschoolers to examine their attitudes toward the UK physical activity and sedentary guidelines for the early years [17]. The results demonstrated a low awareness of the guidelines among the majority of mothers. Although participants felt the guidelines were appropriate for the general population, some mothers felt the guidelines were unnecessary; they believed their children were already meeting the physical activity and sedentary behaviour recommendations. Additionally, some participants raised concerns that the guidelines could place undue pressure or stress on mothers if they could not provide additional physical activity opportunities for their children due to time constraints [16].

In Canada, parents of children who were younger than 4 years and were enrolled in child care were invited to participate in semi-structured focus groups to examine parental perceptions of the new *Canadian Sedentary Behaviour Guidelines for the Early Years* [18]. Overall, there was support for the content of the guidelines and participants found the information clear and helpful. However, there were concerns that the guidelines ‘lumped’ together all sedentary activities (including activities like colouring and reading) as ‘bad’. Previous research also suggests that the guidelines could be a source of guilt among parents, in light of demanding family obligations, the omnipresence of screens, and cold weather conditions [17]. Overall these studies suggest that although parents support and value the development of guidelines for physical activity and sedentary behaviour for their children’s early years, meeting the recommendations in their totality may be challenging.

In June 2016, the first *24-Hour Movement Guidelines for Children and Youth (5–17 years)* were released in Canada [19]. These novel guidelines encompassed three movement behaviours: physical activity (light, moderate, and vigorous); sleep; and sedentary behaviours within a 24-h period. In response to the development of these guidelines, Faulkner et al. [20] conducted a study examining stakeholders’ perceptions of the *24-Hour Movement Guidelines for Children and Youth* through semi-structured focus groups with 104 stakeholders including parents, teachers, exercise professionals, paediatricians, and youth. Overall, there was consistent support across stakeholder groups for the guidelines, with the exception of youth participants, who did not consider their future health as immediately relevant. Stakeholders identified a range of barriers to the uptake of the guidelines including concerns with accurately defining key terms such as ‘recreational’ screen time; everyday financial and time constraints; and the possibility of the guidelines becoming another source of stress and guilt for busy and overwhelmed parents. This notion of parental ‘guilt’ is a recurring theme within studies examining perceptions of national guidelines.

The current study, which was conducted concurrently with the development of the Canadian 24-Hour Movement Guidelines for the Early Years (0–4 years) [21], was designed to replicate the process described by Faulkner and colleagues [20] and had the following objectives: (1) to explore stakeholder (experts in pediatric and family medicine, physical activity knowledge translation, and child care) and end user (parents and child care professionals) perceptions of the Movement Guidelines, and (2) to identify their acceptability, perceived barriers to implementation, and recommended methods and credible messengers of dissemination.

## Methods

Data collection for this study involved two distinct phases. In phase 1, telephone interviews were conducted with leading stakeholders from a range of relevant fields (e.g., pediatric medicine, knowledge translation, and child care). In phase 2, focus groups were conducted with primary end users including parents and practising early childhood educators (ECEs).

### Phase 1: Data collection interviews

Key stakeholders who worked with children 0–4 years of age were purposefully recruited for their expertise from the areas of pediatric and family medicine, early childhood education, physical activity communication, and early childhood physical activity research to participate in telephone interviews. Four of the stakeholders were members of the research panel who contributed to the creation of the Canadian 24-Hour Movement Guidelines for the Early Years (“Movement Guidelines”), four were recruited through snowballing techniques, and the remaining two were identified through an online search for ECEs representing national and local organizations.

Participants were provided with a plain-text draft of the Movement Guidelines [21] by email and subsequently asked in a telephone interview about their first impressions of the guidelines, challenges and barriers to implementing the guidelines, and methods and messengers for dissemination. Given that key stakeholders were selected because of their likelihood to be messengers for the Movement Guidelines, interviews concentrated on gathering specific dissemination strategies (e.g., What are the best ways to communicate the new guidelines to your particular constituency? How is information shared within your professional network? What resources do you need as a [interviewee’s job title] in order to provide information about the guidelines to parents?). Interviews ranged between 25 and 47 min, and were conducted between February and March 2017.

Ethics approval was obtained from the University of British Columbia Research Ethics Board. All of the telephone interviews were held in English, and were conducted by one researcher (Ramanathan). Informed consent was obtained in English at the beginning of the interview, and all interviews were audio-recorded and transcribed verbatim.

#### Participants

A total of 10 key stakeholders consisting of physicians (1 family physician, 2 paediatricians), ECEs in administrative positions with field experience (2), physical activity communicators (1 knowledge exchange, 1 advocacy, 1 education), and researchers (2) participated in telephone interviews. In the end, there was representation from British Columbia, Alberta, Ontario and Quebec and a national organization. Eight of the ten interviewees were female.

### Phase 2: Data collection focus groups

Focus groups were chosen for data collection in this phase as they are useful in identifying shared experiences and knowledge among participants, helping to stimulate further conversation, and identifying contrasting perspectives on the same topic [22–24]. ECEs, ECE trainees, and parents with children 0–4 years were purposefully recruited through existing networks. For example, parents were recruited through activity programs at the University of British Columbia and through local community centres. ECEs were recruited through University of British Columbia Child Care Services, Simon Fraser Child Care Services, local child care centres in Vancouver, the Early Childhood Education program at the University of British Columbia, and the Children’s Hospital of Eastern Ontario. Each focus group was composed of participants of similar background (e.g., a focus group of all parents or all ECEs). At the beginning of each focus group, informed consent was obtained (in either English or French), followed by a short demographic survey. Ethics approval was obtained from the respective institutional Research Ethics Boards in Ontario and British Columbia, where focus groups were held.

Semi-structured focus group guides were developed for parents and ECE end users (available from the corresponding author on request). At the beginning of each focus group, participants were asked about their awareness of the current Canadian Physical Activity [25] and Sedentary Behaviour [26] guidelines for the early years. Participants were provided with a plain-text printed draft of the Movement Guidelines [21] and the opportunity to familiarize themselves with the document. Participants then engaged in open-ended discussions about their first impressions of the Movement Guidelines, the clarity of the guidelines, and the need for an integrated guideline (e.g., Do you find these integrated guidelines helpful or not helpful?). Participants were also asked to provide feedback regarding the specific wording of the Movement Guidelines (data not reported here). They were then invited to discuss the compatibility of these guidelines for daily life and the work environment (e.g., How practical do you think these guidelines are for your work?); the challenges and barriers to implementing the guidelines (e.g., Are there any barriers to implementing these guidelines?); and the best methods and messengers for disseminating these guidelines (e.g., What are the best ways to present or communicate these guidelines? Who would be the best individuals to provide information to you about the guidelines?). Probes were used to encourage discussion throughout the focus groups.

Three authors (Faulkner, Riazi, O’Neill) led the focus groups and interviews in British Columbia, and an additional researcher was hired to lead the francophone focus group in Ontario. Two of these three authors were present at each focus group in British Columbia, with one researcher leading the focus group and the other assisting in taking notes and follow-up questions. A single researcher led and took notes during the francophone focus group in Ontario. All focus groups were audio-recorded and transcribed verbatim.

#### Participants

A total of 92 end users consisting of parents, ECEs, and ECE trainees participated in focus groups. Fourteen focus groups were conducted with parents (*n* = 6), ECEs (*n* = 7) and ECE trainees (*n* = 1), with the number of participants per focus group ranging from 3 to 10. Three participants were interviewed due to scheduling conflicts that prevented them from attending a focus group (representing two additional meetings with end users). Focus groups and interviews were conducted from March to April 2017 in Vancouver and Burnaby (British Columbia) and Orléans (Ontario). Focus group meetings ranged from 40 to 95 min.

End users consisted of parents (*n* = 40), ECEs (*n* = 48), and ECE trainees (n = 4), with the majority of participants (83%) being female. The age of end users ranged from 23 to 68 years, with an average age of 39 years. Among parents, the average number of children per household was 1.8. Most participants self-identified as Caucasian (38%) or Chinese (17%). The rest of the participants represented a diverse mix including Aboriginal, European, South East Indian, Hispanic, African, Portuguese, Filipino, Brazilian, Korean, Iranian, Egyptian, Greek, and Hungarian. The majority (82%) of end users had completed a post-secondary education. Participants received a small remuneration for their participation in the focus group.

### Analysis of interviews and focus groups

Audio-recordings from the interviews and focus groups were transcribed verbatim. To maintain confidentiality, participant names were replaced using the following designations: Parent, Early Childhood Educator, Physician, Physical Activity Communicator, and Researcher. Participants (male = M; female = F) partook in either a focus group (FG) or interview (I) conducted in either English (E) or French (F) languages. Therefore, the designation Early Childhood Educator;M;FG;E would refer to a male Early Childhood Educator who participated in an English focus group.

We replicated the analytic process described by Faulkner et al. [20]. Analysis of the transcribed documents followed an established protocol for thematic analysis [27]. QSR International’s NVivo 11 Software was used to facilitate qualitative data analysis. The transcripts were read line by line, and inductively coded for analysis. Using constant comparison, each coded piece of data was taken and compared to ones preceding it and then categorized into themes [28]. Then relationships between these higher-level concepts were determined through analysis and grounded in the data [29]. These higher-level themes form the basis of the results section.

A number of criteria were adopted to enhance the “trustworthiness” of this study. First, the focus group and interview transcripts were coded independently by two authors (Riazi and Ramanathan). This analysis independently confirmed the primary outcome themes generated. Second, throughout the analysis process, interpretations were also shared and discussed within the broader research team to challenge the identified themes and their connections in a form of peer debriefing [30].

## Results

### Receptivity: ‘The whole picture’

There was consistent support for the new Movement Guidelines across all stakeholder interviews and end user focus groups. Participants demonstrated receptivity to the guidelines regardless of their cultural, educational, or professional backgrounds (e.g., parent, ECE, physician, etc.). Across all interviews and focus groups, participants applauded the clarity and conciseness of the guidelines. As one stakeholder (Researcher;F;I;E) explained, “I thought they were fairly clear, concise, and systematic in their presentation”. Participants liked that the information was divided based on age (i.e., infants, toddlers, and preschoolers), making it easier to access. One participant (Physical Activity Communicator;F;I;E) pointed out that “breaking it up into the 3 categories makes it more usable for people working in this area”. Parents and ECEs agreed that the separation of age made it easier for them to locate the information pertinent to them.

Discussion around the utility of the guidelines revealed that well-received components of the Movement Guidelines included the provision of specific and concrete goals, the inclusion of sleep and quality sedentary activities (e.g., reading, drawing), and the recommendation for replacing indoor with outdoor time. ECEs in particular appreciated the inclusion of sleep (particularly naps) in the guidelines because it was an important component of their child care programs. One ECE (Early Childhood Educator;F;FG;E) explained that having a guideline that incorporates sleep helped to support the advice she gave to parents:

*I think the actual existence of it when it’s completed would be really helpful for me because often, parents ask over the years, “How long should my child be sleeping?” or “What do you think?” and we give educated guesses. To me, it’s helpful to have something to refer to or to send parents to.*

The recommendation for trading indoor for outdoor time was also well received by participants. Some child care providers reported feeling resistance from parents when taking children outside in poor weather conditions (e.g., rain), and felt that the Movement Guidelines would help support dedicated outdoor time within their programming. In addition, the majority of child care providers felt they were already meeting most of the recommendations for sleep (e.g., naps), physical activity, and minimal screen time as part of their licensing regulations. For parents, the Movement Guidelines provided a goal to strive toward within their homes.

Additionally, participants liked the integration of the three movement behaviours (i.e., sleep, physical activity, and sedentary behaviours) in a single guideline. Participants liked the holistic nature of the guidelines, which encompassed movement behaviours during a 24-h period. The integration helped raise awareness that “all the components are important” (Parent;F:FG;E). One father (Parent;M;FG;E) discussed the benefit of following integrated guidelines:

*Yeah, if [children are] really physically active, they sleep better; they’re tired at the end of the day. They’re more likely to sleep throughout the night. If they’re sedentary, they’re more irritable I suppose. It all plays together.*

Similar to other participants, a researcher (Researcher;M;I;E) agreed that a 24-h perspective for the Movement Guidelines was useful:

*My first impression was that it was a good idea to go to the 24-hour movement approach. It makes a lot of sense from a public health messaging perspective to deliver the messaging in the context of what a healthy day looks like. This is an advantage of the current guidelines.*

The majority of stakeholders recognized the relationship and interplay between the movement behaviours as well as the importance of the individual behaviours. Many also wanted and expressed a need for such guidelines. For example, researchers felt that they could use the Movement Guidelines as measurable targets in their studies, and physical activity communicators felt that the recommendations would enhance the structure and add a level of integrity to their workshops. The guidelines provided an evidence-based resource for stakeholders in medicine and early childhood education, while also providing parents with a set of recommendations to work toward.

### Barriers to uptake

While participants showed receptivity toward the proposed Movement Guidelines, barriers were discussed in regard to the uptake of the guidelines. Three themes were identified that encompassed potential barriers to adoption of the guidelines. These were: a lack of information about the guidelines; ‘daily challenges’ such as the attraction of screen time, lack of time, and competing priorities; and the challenging nature of current social norms.

#### What guidelines? Lack of awareness among parents and educators

Awareness of the existing guidelines for physical activity and sedentary behaviour was low among focus group participants. The majority of parents were not aware of the physical activity and sedentary behaviour recommendations, with only a small portion recalling some information about sleep recommendations. Some parents recalled hearing about sleep recommendations from their child’s physician or hearing physical activity-related messaging for children and youth through television ads and social media. However, the vast majority of focus group participants could not remember ever seeing the existing physical activity and sedentary behaviour guidelines, and often were not aware of where to access them. As one stakeholder (Researcher/Parent;F;I:E) explained:

*I’ve been doing work trying to increase education around movement in child care centres. I’ve realized over the last 5 years or so that a lot of child care staff aren’t currently aware of our present physical activity guidelines for the early years.*

Although child care providers self-reported meeting the majority of recommendations from the Movement Guidelines in their work, it was by virtue of licensing requirements and the philosophy of the child care centre, and not because they were aware of them. For example, one early childhood educator (Early Childhood Educator;F;FG;E) explained, “Everything, what is written [in the Guidelines], it’s happening right now in my daycare, so this is nothing new to me”. A future challenge in raising awareness of and implementing the Movement Guidelines stems from the variance in policies at child care centres. As one individual (Researcher/Parent;F;I;E) pointed out:

*Each province has a different requirement around outdoor play and physical activity. If the majority of our kids are enrolled in these centres, then that will have a large role in whether the kids are sufficiently meeting these guidelines.*

There is also variation among child care providers in regard to whether or not they are licensed by the province. For instance, unlicensed (licence-not-required) home child care providers may care for only two children or a sibling group at any one time and do not have requirements for meeting any specific standards for health or safety (e.g., no regulations for screen time) [31]. On the other hand, licensed child care providers provide care for three or more children and must meet specific requirements related to health, safety, space, staffing qualifications, and program standards. Additionally, variation exists amongst types of licensed child care providers. For instance, some occasional child care centres (e.g., provide only part-time or occasional care) may not have the capacity to provide outdoor space for physical activity in comparison to a full-time child care centre [31]. Consistent adoption of the Movement Guidelines will require a focus on policy changes or licensing regulations in child care centres as well as providing support for implementation.

#### ‘Daily challenges’

Although participants demonstrated receptivity toward the new Movement Guidelines, they also identified daily challenges such as the allure of screen time, lack of time, and competing parental responsibilities. The most commonly mentioned challenge was screen time – referred to as a “hot button issue” by one parent (Parent;F;FG;E). Although most parents felt they were meeting screen time recommendations during the week, many admitted that screen time over the weekend often exceeded the recommendation of one hour or less. There was also some confusion about what qualified as screen time. Parents tended to rationalize screen time positively if it was interactive, such as completing a puzzle on a tablet. One father (Parent;M;FG;E) explained:

*The four-year-old has a lot of iPad time, but it’s all very much school-based learning. Either a puzzle or math programs or things like that. As far as time goes, it’s way exceeding it, but we’re trying to at least...it’s not watching TV all day. It’s doing something at least interactive to help stimulate cognitive function. That’s how I rationalize it.*

At the same time, the majority of stakeholders and end users understood the potential detrimental effects of extended periods of screen time for children. Parents and child care providers discussed the consequences of prolonged screen time, including children being more lethargic, less inclined to engage in physical activity, and more irritable. Parents, however, also discussed the use of screens as a bargaining tool or reward if their child completed a certain task (e.g., finishing their dinner) or as a “babysitting device” (Parent;M;FG;E) while the parent was cooking dinner or shopping. One father (Parent;M;FG;E) explained that every day is different:

*Every day you’re not going to hit it [meet the guidelines]. You’re choosing battles. You’re out trying to shop and your kid is screaming and doesn’t want to stay in the shopping cart. You give them your phone to keep them entertained for 15 minutes while you do whatever.*

ECEs, on the other hand, stated that they were meeting the recommendations for screen time and outdoor play. All child care providers explained that their centres had policies that stipulated either minimal or zero screen time for children under their care, as well as dedicated outdoor play time regardless of weather. Due to the time children spend in child care, these centres represent an ideal avenue to help children meet the recommendations for screen time and physical activity, at least while children are in their care.

Lack of time and competing responsibilities were common challenges among parents who expressed concern over their children meeting the guidelines. Consequently, a number of participants agreed that the guidelines could be a source of guilt for parents. The concern was that the guidelines could become “one more thing, like one more guideline, or one more 180 minutes” for parents to worry about (Early Childhood Educator;F;FG;E). Upon reading the proposed Movement Guidelines, one parent (Parent;F;FG;E) stated,

*I remember reading something along the lines of being – like [the guideline for a child] not being restrained for more than an hour at a time, and then thinking about how like just driving up to Whistler takes like an hour and a half or two hours with the traffic, and then I think I read this and I think I cried again because I was like, “oh, no, she was restrained in the car seat for two hours! I’m a terrible mother!”*

However, other parents countered that it was important to remember that the Movement Guidelines were recommendations only. “I think it’s a good reminder – it’s goals, right? But you’re not going to reach this every day” (Parent;F;FG;E). The majority of parents agreed that the guidelines were important goals to strive toward, but that realistically, they might not meet them every day given household chores. One mother (Parent;F;FG;F) expressed the need to face “reality”:

*Say that I have to clean the house, it’s [screen time] sometimes the only way to capture their attention. It gives them something to watch. […] Yeah, the government should send a housecleaner. Someone to prepare dinner. It’s [screen time] just a way to distract them, because we have too much on our plate, we work full-time, we have to clean the house and do chores, our houses are becoming bigger now… it’s a lot.*

Physical activity in the older age groups (toddlers and preschoolers) raised some concerns among parents who found 180 min of physical activity including energetic play to be overwhelming. However, after clarifying that the 180 min could be accumulated throughout the day, parents felt that the recommendations were achievable. One stakeholder (Physical Activity Communicator;F;I;E) explained that one needs to make “connections to other things so that physical activity is not at the expense of something else. If it is integrated into your healthy, active day, then it is easier to achieve”. Even when a parent feels tired, said one stakeholder (Researcher/Parent;F;I;E), it is important to incorporate physical activity in daily life without it feeling like an ‘add-on’:

*Recognition and education around how activities can be embedded within a day is important. It doesn’t have to be a set 60 minutes for going outside and playing; it doesn’t have to be that structured…Trying to think about creative ways to embed it within their daily activities that are already happening, so it doesn’t feel like an add-on, but rather a build-in, will be helpful.*

In contrast, a few focus group parents felt that their children were exceeding the recommended levels of activity. One parent (Parent;F;FG;E) said, “Mine never stop moving, so I’ve never considered there being any guidelines. They have so much energy”. At the same time, interviewees who were engaged in physical activity advocacy and research noted the issue of overestimation: “Sometimes people will just break it down into active or not active, and we tend to overestimate how active we are in retrospect and how active kids are” (Physical Activity Communicator;F;I;E).

#### Shifting social norms

Several participants explained that as a society, we may need to shift our social norms in order to successfully implement the Movement Guidelines, particularly for screen time and energetic play. Screens and technology were described as ubiquitous in today’s society, and respondents said they were used as rewards and substitute babysitters for even the youngest children. As one researcher (Researcher;M;I;E) pointed out, “It is socially acceptable to be using screens. Parents may feel like they are not good parents if they are not providing the latest technology to their child”.

With respect to recommendations for energetic play, physicians, parents, ECEs and physical activity communicators noted that there are competing priorities within families and child care settings, where physical activity often gets pushed aside. A physician (Physician/Parent;F;I;E) explained that she often sees “patients that have culturally placed an emphasis on reading and achievement in the preschooling group. An increasing focus is placed on learning instead of the importance of energetic play”. ECEs offered the perspective that as a society, we have moved away from “a culture of the children being participants in the house” (Early Childhood Educator;F;FG:E) where they can accumulate physical activity by helping with physically demanding chores. Children in today’s society are so busy in structured programs and activities, even as preschoolers, that parents claim there simply is not enough time for active play. To meet recommendations for energetic play, greater value will need to be placed on free play and play-based learning once again:

*The focus needs to be on simple play – that’s where you build your pre-math, social relationships, and healthy habits. It really just has to be a simple reminder to families and to anybody that works with young kids that it is just about play. Let’s not over-think this! It’s not that hard to get kids actively involved. (Early Childhood Educator;M;I;E)*

### Messengers and methods of dissemination

Participants shared a number of ideas on how to present and communicate the Movement Guidelines to Canadian families. In this section, suggestions for dissemination have been organized into settings (location), existing communication channels (mechanisms), and media (format).

#### Settings for dissemination

All parents and professionals agreed that information about the Movement Guidelines should be disseminated to families in a variety of settings to increase opportunities for awareness and uptake. Medical settings (e.g., doctor’s offices, hospitals), child care settings (e.g., daycares, home care), community centres (e.g., libraries, community schools, early years centres), and schools (for four-year-olds) were cited across interviews and focus groups. Particular emphasis was placed on physician visits, partly because promotion of healthy behaviours (such as physical activity and adequate sleep) was viewed as the “business” of physicians, and partly due to the frequency of visits among Canadian infants and preschoolers to receive publicly funded immunizations. Physicians also felt that it was their responsibility to discuss the importance of energetic play, adequate sleep, and minimizing screen time as part of preventive health care that promotes healthy growth and development.

Child care settings were seen as another natural conduit for sharing the Movement Guidelines as these settings serve families with infants, toddlers, and preschoolers. ECEs and daycare staff generally said they endeavoured to build trusting relationships with families, often shared information about activities and initiatives that could be reinforced at home (e.g., literacy techniques), and considered themselves as having a “huge role in child development” (Early Childhood Educator;F;FG;E). One ECE (Early Childhood Educator;F;FG;E) said:

*I feel like our role is mostly liaising with the parents. Maybe trying to notice where there might be gaps and knowledge of what’s needed at that age and volunteering information when it might be useful.*

In this way, the Movement Guidelines could easily be integrated within child care programming, with notices sent home and verbal reinforcement given during meetings with parents. Many participants working in child care settings felt that their centres already adhered to the guidelines for energetic play. In these instances, the guidelines could be used to justify current practices of encouraging energetic play outdoors in inclement weather such as rain and snow: “Some parents would say, ‘Oh, why are they playing outside so much?’ [And with these guidelines, we could respond with] ‘They’re playing outside because you know what, you’re supposed to… this is one of the national guidelines…” (Early Childhood Educator;F;FG;E).

With respect to community centres across Canada, it was suggested that programming for infants, toddlers, and preschoolers with caregiver accompaniment (e.g., Strong Start in British Columbia, Ontario Early Years programs in Ontario) and programming for preschoolers (without caregiver accompaniment) could introduce and model the Movement Guidelines. Like physicians, ECEs recognized the important role they played in sharing health and wellness information, and were keen to play their part:

*Female ECE (Early Childhood Educator;F;FG;E): Preschool teachers…have so much clout and so much…*

*Interviewer: They hold influence…*

*Female ECE: …influence. I never actually realized it until…I’m only, I’m new as a Strong Start facilitator, so only a couple of years, and it’s just amazing how much parents really look to you.*

Repeatedly hearing about the Movement Guidelines from credible sources in a variety of settings would be likely to prompt action and to foster awareness and mindfulness among families. For example, one parent (Parent;F;FG;E) explained, “…Getting those reminders all the time I think is a good thing because it’s easy to forget and then you get into kind of these routines, and maybe bad habits let’s say, and so just to have that constant reminder I think would be a good thing”. There was also a sense that in order to promote uptake, there would need to be an endorsement of the guidelines from professional societies, such as the College of Family Physicians of Canada, the Canadian Paediatric Society, and the Public Health Agency of Canada. These endorsements, which would ultimately enhance the credibility of the Movement Guidelines, could take the form of logos integrated within the Movement Guidelines, or web links to the guidelines posted on the websites of professional societies and government organizations.

#### Disseminating the movement guidelines within existing communication channels

With many settings and programs already devoted to the early years, there was consensus that the dissemination and, to some extent, the implementation of the Movement Guidelines should be integrated within existing communication channels. For instance, adherence to the guidelines could become a licensing regulation for child care programs. As another example, pre- and post-natal classes at hospitals and public health units could introduce new parents to the Movement Guidelines in their sessions: “The guidelines could be embedded within prenatal care, to motivate people to start thinking about how to structure the day before even having children” (Researcher;M;I;E). With respect to post-natal care, a physician explained that nurses in Ontario already deliver a Healthy Babies, Healthy Children public health program with home visits or telephone calls for new parents, and information about the Movement Guidelines could be included in these visits. Non-emergency health information from registered nurses is also available to parents and caregivers through telephone help lines such as Telehealth in Ontario. Information about the Movement Guidelines could be made “…available to nurses who are going into homes of moms with babies and helping them with sleep, breastfeeding, or formula feeding. The guidelines can also be part of the information that is going to moms, and nurses may [even] model the appropriate behaviours” (Physician;F;I;E).

A physical activity advocate (Physical Activity Communicator;F;I;E) also suggested that existing workshops for parents could be updated to include the recommendations:

*Public health units are amazing and they generally have a physical activity promoter on staff, if not more than one, and also a child and family health manager. They have a mandate to do workshops and to connect with parents. Those would be good avenues [for dissemination].*

Other mechanisms cited were educational programs for newcomers as a part of settlement services; government mail-outs and electronic notices (e.g., notifications about the Children’s Fitness Tax Credit); and listservs and newsletters (e.g., from daycares, community centres, health professional organizations).

#### Media for dissemination

Focus group and interview participants cited traditional media (e.g., television ads, print news, radio), social media (e.g., blogs, Facebook, Twitter) and websites as key media to disseminate the Movement Guidelines to families. Parents and professional participants favoured electronic media because of the potential to view video and other visual examples of the Movement Guidelines (e.g., infographics), interact with the information (e.g., click on a link for further information), and easily share information personally and professionally. Web and mobile apps were also identified as important electronic mechanisms to learn about and track the guidelines. Parents who used pregnancy apps to track fetal growth, and later used breast/bottle-feeding apps to track infant feeding and development, saw the potential for apps to track energetic play, sleep, and sedentary behaviour. These apps would also serve to alert parents about the Movement Guidelines and provide suggestions for achieving them as their child grew from infant to preschooler. In medical settings, most clinicians (e.g., nurses, physicians, physiotherapists) have access to mobile apps and view apps as “a rapid way of knowing what the guidelines are and ideally implementing them in a clinical setting” (Physician;F;I;E). It was suggested that apps could be used to prompt clinicians to screen for behaviours, feed into electronic medical records, or even serve as screening tools for parents to complete while sitting in waiting rooms. One physician explained, “If parents don’t prioritize these behaviours on their own, they would not necessarily ask their physician about it. It is up to the physician to screen for them and begin the discussion” (Physician/Parent;F;I;E).

Participants working in physical activity promotion also used electronic media to promote programs and initiatives. One participant (Physical Activity Communicator;F;I;E) explained, “In the webinars that we do, we could leverage the new guidelines, and definitely through our communications we can start some conversations around these guidelines”. One final suggestion for disseminating the Movement Guidelines in an electronic format was an online training module for ECEs and primary school teachers. A researcher-informant (Researcher;F;I;E) noted, “The most fiscally responsible time and logical time [to disseminate information about the guidelines] is [physical activity] training within the ECE college curriculum before they enter the field” and that an “online module that ECE programs can pick up and administer to their students prior to leaving the college” was an ideal way of equipping educators with the skills to facilitate energetic play in daycare settings. Such a module could also be useful for ongoing professional development of teachers as well as license renewal for ECEs.

Non-electronic formats to disseminate the Movement Guidelines included printed pamphlets, posters, magnets, professional conference presentations and booths, peer-reviewed journal articles (and their electronic versions), and a telephone help-line. One suggestion from the key stakeholder interviews was the development of ready-to-use-tools such as workshop outlines with practical strategies, discussion points, and activities geared toward ECEs. As explained by a physical activity educator (Physical Activity Communicator;F;I;E), “If there were resources that showed what the guidelines look like in practice in homes, daycares, at a community centre, in a community in general, that would be helpful”. While there was unanimous support for the spirit of the Movement Guidelines among ECEs, they felt a one-hour practical workshop delivered to program facilitators in child care and community settings would alleviate the burden of determining how to incorporate the Movement Guidelines into a curriculum-packed day.

## Discussion

This study examined perceptions of stakeholders (experts in medicine, physical activity knowledge translation, and research) and end users (parents and ECEs) on the new 24-Hour Movement Guidelines for the Early Years (0–4 Years). It is valuable to engage stakeholders and end users in the development of guidelines in order to assess acceptability of information, barriers to uptake, and identification of key messengers and methods for dissemination [15]. Overall, there was consistent support for the Movement Guidelines across all stakeholder and end user groups regardless of gender, cultural background, or profession. The integration of physical activity, sleep, and sedentary behaviour into one all-encompassing guideline was well received. This is an encouraging finding as the uptake of a new innovation is strongly related to its perceived acceptability by potential adopters [32].

The majority of expert stakeholders and end users perceived the Movement Guidelines as providing a set of healthy goals that were largely achievable. In comparison to the *24-Hour Movement Guidelines for Children and Youth* [20], there appeared to be less tension among participants, particularly parents, regarding the likelihood of their child meeting the Movement Guidelines. Parents discussed how their toddlers and preschoolers were ‘naturally’ active. This perception may be accurate, as 70% of Canadian 3- to 4-year-olds meet the daily recommendation of at least 180 min of physical activity at any intensity [33]. Parents also described having control and awareness of their child’s sleep patterns and access to screen time. Additionally, licensing regulations at child care centres were congruent with the Movement Guidelines with respect to nap times, minimal screen time, and scheduled outdoor time. Meeting the Children and Youth Guidelines, on the other hand, raised more concerns regarding feasibility since physical activity, screen time, and sleep in this age group were affected by numerous factors such as school structure (e.g., more time spent sitting), ubiquity of screen time (e.g., video games, screens in school), and time spent doing homework (e.g., may affect hours of sleep in older youths).

Despite the overall acceptability of the Movement Guidelines for the early years, participants cited a number of challenges to uptake. Such challenges were largely centred on discussions about screen time – suggesting that screen time was increasingly creeping into the lives of their children, and was inevitable. In the examination by Carson and colleagues of parental perceptions of the *Canadian Sedentary Behaviour Guidelines for the Early Years* [18], parents also expressed the “need to balance multiple demands of family life” (p. 3) and feelings of guilt when not adhering to the guidelines. For example, in Carson et al. [18] and the present study, parents stated that they used children’s screen time as a tool to finish daily tasks (e.g., meal preparation, household chores) or to reward children for good behaviour. In both studies, parents also discussed the educational value of some television programs and the use of tablets for interactive learning activities. This perception that screens can be used as an educational resource in the early years has increasingly been documented in studies from around the world [17, 18, 34, 35], which demonstrates shifting social norms and value placed on screen technologies, even for our youngest children.

At the same time, parents expressed concerns about screen time and acknowledged the potentially harmful effects based on experience monitoring their child’s behaviour or through intuition. Speculatively, the theory of cognitive dissonance [36] might be one relevant consideration in interpreting reactions to the Movement Guidelines in our study as perhaps in other studies. This theory argues that dissonance is a negative state that occurs when an individual simultaneously holds two cognitions (e.g., attitudes or values) that are psychologically incompatible. Since dissonance is likely unpleasant, one is motivated to reduce it by adding, changing, or reducing the importance of cognitions to make them more compatible. Aronson [37] argued that central to dissonance theory is not the conflict of two opposing cognitions, but a conflict between the self-concept and cognitions about some behaviour. In the current context, a parent highly values the health of their child and may be aware that too much screen time may be harmful. Yet, if the child is engaging in screen time exceeding recommended guidelines, then other cognitions are sought to avoid dissonance – that screen time is educational, chores need to be done, or bad weather is a barrier. This is not to discount these reasons as real challenges for parents, but it is also important to consider the cumulative effects of cognitive dissonance in responding to the Movement Guidelines in general. Following on this theoretical perspective, individuals are motivated to avoid attitude-dissonant information in order to stabilize an existing attitude or decision – or, in other words, turning parents off the guidelines as reported in one study [18]. In our study, the Movement Guidelines were interpreted by parents as ‘goals’ to strive toward, but unrealistic most days. Cognitive consonance is maintained.

This is theoretical speculation, but cognitive dissonance may provide an interesting lens for future research examining perceptions of Movement Guidelines across the lifespan. A particular focus is on screen time, given acknowledged parental concerns over this issue. As others have noted [17, 18], a good starting point is to include messaging about Movement Guidelines that are gain-framed, pragmatic, and include practical strategies for replacing screen time. This will assist with reinforcing confidence that the recommendations are achievable. This should occur within a systematic, multi-level approach including efforts to change how people think about the guidelines [16] and how environments and policies support people to meet those guidelines [38].

Similar to previous research in Canada [20] and the UK [17], the present study found that participants had a low awareness of existing guidelines. Setting cognitive dissonance aside for a moment (i.e. low awareness reflects avoidance of attitude-dissonant information), the likely explanation may be the lack of effective (or of any) dissemination efforts. For example, a lack of a sustained and funded approach to the knowledge translation of the current Canadian physical activity guidelines for adults has been recognized as potentially explaining low awareness of their recommendations [16]. It is important to develop evidence-based guidelines, which act as a basis for planning, policy, and evaluation; however, equally crucial are the efforts to “promote awareness, acceptance, adoption, and adherence to guidelines” (p. 1) [39]. Nevertheless, there appears to be a gap between the development of guidelines and their translation into policy or practice [40, 41]. Future dissemination of the Movement Guidelines, with dedicated funding, will require consideration of the recommendations suggested by participants.

Interactions with the medical system and/or medical professionals were obvious points of contact when information about the Movement Guidelines could be conveyed. Parents also identified child care settings as important for receiving information about the guidelines. In 2011, more than half (54%) of Canadian parents with children under 4 years of age indicated they used child care [42]. Notably, ECEs were accepting of their role as promoters of the Movement Guidelines, and recognized that they played an important part in children’s growth and development. ECEs may therefore be key messengers for communicating the Movement Guidelines to parents, and in helping children meet the recommendations at least while under their care. Disseminating the Movement Guidelines within existing systems of knowledge exchange for medical and child care professionals makes intuitive sense, and will vary by profession and jurisdiction. However, there was agreement among study participants that dissemination and implementation of the guidelines should be a collaborative approach.

The Movement Guidelines need to be disseminated through a number of messengers (e.g., medical professionals, ECEs, Canadian government) and methods (e.g., traditional formats, social media, workshops). While suggested methods of dissemination were wide and varied, there was some consensus that web-based and mobile applications would be useful for prompting discussion of the Movement Guidelines, tracking behaviours, and providing creative, interactive solutions to help implement them. Repetition of the information was considered vital; participants repeatedly mentioned the value of receiving the information from diverse sources at multiple times, in varying formats.

### Strengths, limitations, and future directions

Strengths of this study included a relatively large sample size (*n* = 102) with a professionally diverse range of stakeholders and end users. However, the sample was primarily female (*n* = 84) and from urban settings, with the majority of participants having completed a post-secondary education. An exploration of perspectives of Indigenous parents, for example, and sampling on a broader geographic scale would be informative. Attempts were made to include stakeholders and end users of varying professional backgrounds (e.g., experts in medicine, researchers, parents, and ECEs), but we acknowledge that some important voices have not been considered regarding the acceptability of the Movement Guidelines. Given participants’ recommendations to incorporate the Movement Guidelines into licensing standards and regulations, another voice missing in this study is that of policy-makers. We attempted to recruit a public health policy-maker for the stakeholder interviews, but were unable to do so within the study timelines. Another potential limitation was that four of the stakeholder interviewees had contributed to the creation of the Movement Guidelines. However, as interviews focused on dissemination strategies to professional groups and end users, it was both appropriate and advantageous to include individuals who were already familiar with the Movement Guidelines. One final limitation of this study was that only one researcher was present in the francophone focus groups and was therefore tasked with both facilitating discussion and taking notes to capture both the order of speakers and key points of conversation requiring follow-up questions. Although the moderator was skilled and experienced, it may be ideal to have two researchers present during a focus group to divide these tasks.

## Conclusions

In summary, stakeholders and end users were very receptive to the Canadian 24-Hour Movement Guidelines for the Early Years (0–4 years). The behavioural recommendations were largely considered feasible, although increasing screen time was identified by participants as an emerging concern in the early years. Engaging physicians and ECEs in dissemination efforts may be critical for increasing awareness of the Movement Guidelines among Canadian parents. In turn, uptake by parents will likely be dependent on the messaging and resources created to facilitate implementation.

## Abbreviations

ECE: Early childhood educator
Movement Guidelines: 24-Hour Movement Guidelines for the Early Years (0–4 years)
